# Activation of the IRE1α Arm, but not the PERK Arm, of the Unfolded Protein Response Contributes to Fumonisin B1-Induced Hepatotoxicity

**DOI:** 10.3390/toxins12010055

**Published:** 2020-01-16

**Authors:** Xiaoyi Liu, Enxiang Zhang, Shutao Yin, Chong Zhao, Lihong Fan, Hongbo Hu

**Affiliations:** 1 Beijing Advanced Innovation Center for Food Nutrition and Human Health, College of Food Science and Nutritional Engineering, Beijing Key Laboratory for Food Non-thermal Processing, China Agricultural University, Beijing 100083, China; liuxiaoyi0602@cau.edu.cn (X.L.); bs20163060470@cau.edu.cn (E.Z.); yinshutao@cau.edu.cn (S.Y.); zhaoch0206@cau.edu.cn (C.Z.); 2 College of Veterinary Medicine, China Agricultural University, No. 2 Yunamingyuan West Road, Haidian District, Beijing 100193, China; flh@cau.edu.cn

**Keywords:** Fumonisin B1, endoplasmic reticulum stress, IRE1α, oxidative stress, hepatotoxicity, autophagy

## Abstract

Previous studies by us or others have shown that endoplasmic reticulum (ER) stress was activated by fumonisin 1 (FB1) exposure, which is considered to be a critical event in the FB1-induced toxic effect. However, the detailed mechanisms underlying FB1-induced ER stress-mediated liver toxicity remain elusive. The objectives of the present study were designed to address the following issues: (1) the contribution of each arm of the unfolded protein response (UPR); (2) the downstream targets of ER stress that mediated FB1-induced liver toxicity; and (3) the relationship between ER stress and oxidative stress triggered by FB1. We also investigated whether the inhibition of ER stress by its inhibitor could offer protection against FB1-induced hepatotoxicity in vivo, which has not been critically addressed previously. The results showed that the activation of the IRE1α axis, but not of the PERK axis, of UPR contributed to FB1-induced ER stress-mediated hepatocyte toxicity; the activation of the Bax/Bak-mediated mitochondrial pathway lay downstream of IRE1α to trigger mitochondrial-dependent apoptosis in response to FB1; FB1-induced oxidative stress and ER stress augmented each other through a positive feedback mechanism; tauroursodeoxycholic acid (TUDCA)-mediated ER stress inactivation is an effective approach to counteract FB1-induced hepatotoxicity in vivo. The data of the present study allow us to better understand the mechanisms of FB1-induced hepatotoxicity.

## 1. Introduction

The endoplasmic reticulum (ER) is a vital organelle and plays a pivotal role in protein synthesis, folding and maturation [1,2]. ER stress can be induced in response to many cellular perturbations, such as oxidative stress, in turn activating an evolutionary conserved signaling pathway named the unfolded protein response (UPR) [3,4,5]. The main aim of UPR is to counteract ER stress through the inhibition of protein translation, enhancing its ability for protein folding and accelerating protein degradation, and to restore ER homeostasis [4,6,7]. However, the excessive or persistent induction of the UPR may change its initial function from adaptation to cell death induction, which has been shown to contribute to the pathogenesis of various diseases, including liver diseases [8].

Mycotoxins are toxic secondary metabolites of fungi and the common toxic substances found in food [9]. It has been well documented that mycotoxin can produces numerous health problems for human and animals [10,11]. Fumonisins, synthesized mainly by *Fusarium verticillioides* and *Fusarium proliferatum*, are a group of mycotoxins often found in maize and maize-based foodstuffs [12,13]. Among them, fumonisin B1 (FB1) is a key member with numerous adverse health effects, including hepatotoxicity. Previous studies by us or others have shown that treatment with FB1 can trigger ER stress, which contributed significantly to the FB1-induced toxic effect [14,15]. However, the detailed mechanisms underlying FB1-induced ER stress-mediated liver toxicity remain largely unknown. The objectives of the current study were designed to decipher the mechanisms involved in ER stress-mediated liver toxicity by addressing the following issues: (1) the functional role of each branch of the UPR signaling; (2) the downstream molecules of ER stress that contributed to FB1-induced liver toxicity; and (3) the relationship between ER stress and oxidative stress induction by FB1. In addition, the possibility of ER stress as a target to counteract FB1-caused hepatotoxicity in vivo has been evaluated, something which has not been critically addressed previously.

## 2. Results

### 2.1. Activation of IRE1α but not the PERK Axis of ER Stress Response Contributes to FB1-Induced ER Stress-Mediated Hepatocyte Toxicity

**Figure 1:**
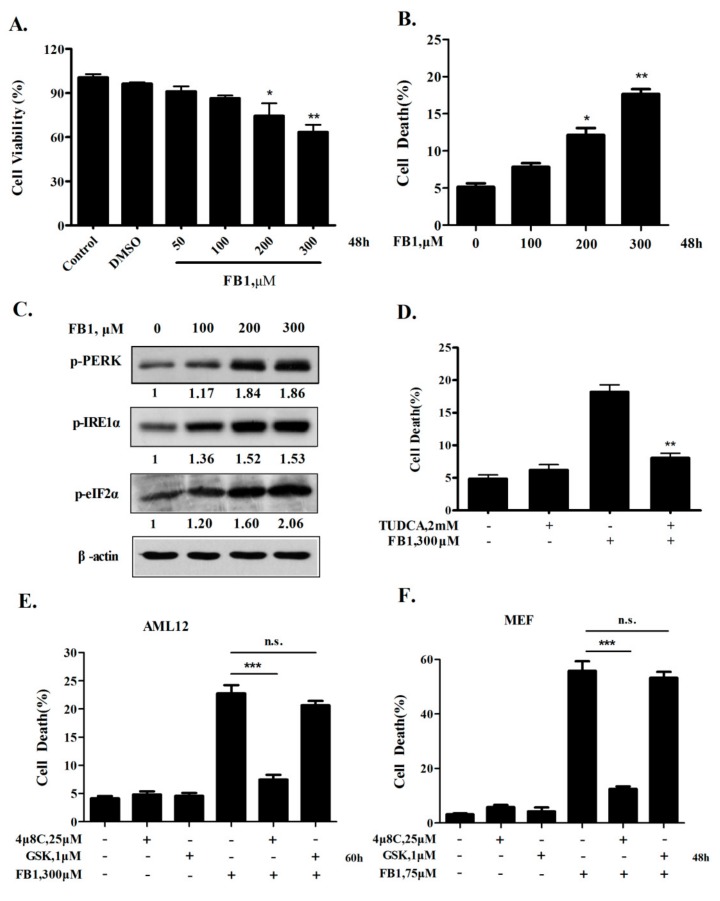
Activation of the IRE1α axis, but not PERK axis, of the ER stress response contributed to FB1-induced ER stress-mediated hepatocyte toxicity. AML12 cells were treated with 50 to 300 μM FB1 for 48 h, and then (**A**) the inhibitory effects of FB1 on the AML12 cells was evaluated by crystal violet staining, and (**B**) the cell death was analyzed by Annexin V/PI staining. (**C**) The effect of FB1 on the ER stress makers, the cells were exposed to FB1 for 48 h, and the phosphorylation of PERK, IRE1α, and eIF2α were analyzed by Western blot. n = 3 (**D**) The influence of the ER stress inhibitor TUDCA on the cell death induction by FB1. (**E** & **F**) The effect of IRE1α or PERK specific inhibitor on FB1-induced cell death in AML12 cells and MEF cells. The cells were exposed to FB1 in the presence or absence of inhibitors for the indicated time, and the cell death was evaluated by the flow cytometry analysis of Annexin V/PI positive cells. The bars denote standard errors from three experiments. * *p* < 0.05, ** *p* < 0.01, *** *p* < 0.001 compared with the corresponding control.

Most existing studies have employed HepG2 liver cancer cells to investigate the hepatotoxicity of FB1 [15]. The cancer attribute poses a key limitation of this cell line as a model for hepatotoxicity study. The AML12 (alpha mouse liver 12) cell line was established from the hepatocytes of a mouse. Given its non-tumorigenic feature, the AML12 cell line may be a more suitable cell model for hepatotoxicity study. AML12 cells were exposed to increasing concentrations of FB1 for 48 h, and cell proliferation and apoptosis were assessed by crystal violet staining and Annexin V/PI staining, respectively. As shown in Figure 1A,B, the FB1 exposure induced a dose-dependent reduction of the cell proliferation and an increase of the cell death induction in AML12 cells. Next, we examined whether ER stress was induced by FB1 in AML12 cells, and the results are shown in Figure 1C. A Western blot analysis of key ER stress markers demonstrated that FB1 treatment caused a dose-dependent increased phosphorylation of IRE1α, PERK, and eIF2α, indicating that ER stress was activated by FB1 in AML12 cells. To determine the role of ER stress in FB1-induced apoptosis in AML12 cells, we tested the influence of ER stress inactivation by its inhibitor tauroursodeoxycholic Acid (TUDCA) on apoptosis induction by FB1. As shown in Figure 1D, FB1-induced apoptosis was significantly attenuated by ER stress inhibition. These results suggest that the AML12 cell line is a reasonable system for addressing the detailed mechanisms of the ER stress-mediated liver toxicity of FB1.

It has been shown that PERK and IRE1α are the two key branches of the ER stress response associated with ER stress-mediated apoptosis [3]. To decipher the contribution of each branch to FB1-inducd apoptosis in liver cells, we evaluated the influence of the specific inactivation of PERK or IRE1α on apoptosis induction by FB1 in AML12 cells. 4μ8C [16] and GSK2606414 [17] were used to specifically inhibit IRE1α and PERK respectively, and apoptosis was measured by Annexin-V/PI staining. As shown in Figure 1E, FB1-induced apoptosis was significantly suppressed in the presence of 4μ8C but not of GSK2606414 in AML12 cells. Similar results were also found in mouse embryonic fibroblast (MEF) cells (Figure 1F). These data suggested that the activation of the IRE1α pathway but not of the PERK pathway contributed to FB1-induced hepatocyte apoptosis.

### 2.2. IRE1α-Mediated Activation of Mitochondrial Pathway Plays an Important Role in Apoptosis Induction by FB1 in Liver Cells

**Figure 2:**
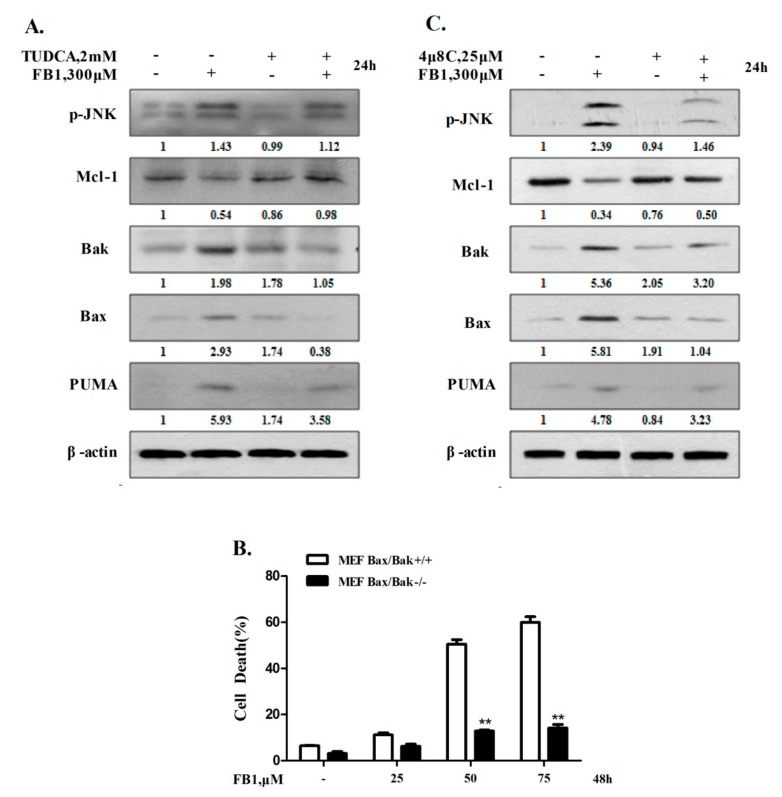
The IRE1α-mediated activation of the mitochondrial pathway plays an important role in apoptosis induction by FB1 in liver cells. (**A**) The effect of FB1 on the expression of JNK, Mcl-1, Bak, Bax, and Puma in the protein level. The cells were exposed to FB1 with or without TUDCA for 48 h, and the phosphorylation of JNK, Mcl-1, Bak, Bax, and Puma were analyzed by Western blotting. n = 3. (**B**) FB1 significantly induced cell death in wild-type MEF cells but not in Bax/Bak knockout MEF cells. The bars denote standard errors from three experiments. (**C**) The effect of the IRE1α specific inhibitor 4μ8C on the expression of apoptosis-related proteins. The cells were exposed to FB1 with or without 4μ8C for 24 h, and the phosphorylation of JNK, Mcl-1, Bak, Bax, and Puma were analyzed by Western blotting. n = 3. ** *p* < 0.01 compared with the corresponding control.

To investigate the downstream molecules of ER stress that mediated FB1-induced apoptosis in liver cells, we examined the effect of ER stress inhibition on FB1-induced apoptosis-related proteins by Western blot analysis. As demonstrated in Figure 2A, FB1 treatment resulted in increased JNK phosphorylation, the down-regulation of anti-apoptotic Bcl-2 family protein Mcl-1, and the up-regulation of pro-apoptotic Bcl-2 family protein Bak, Bax, and PUMA in AML12 cells. To critically determine the role of Bax/Bak in FB1-induced apoptosis, wild-type (WT) mouse embryonic fibroblast (MEF) cells and Bax/Bak double knockout (KO) MEF cells were employed to compare apoptosis induction in these two cell lines. As demonstrated in Figure 2B, FB1 caused a concentration-dependent apoptosis in WT-MEF cells, which was dramatically decreased in Bax/Bak KO-MEF cells, suggesting Bax/Bak played a pivotal role in FB1-induced apoptosis. In line with the protective effect of IRE1α inhibition on apoptosis induction, the FB1-induced changes of apoptosis-related proteins were ameliorated in the presence of the IRE1α specific inhibitor 4μ8C (Figure 2C), further supporting a pivotal role of IRE1α in FB1-induced hepatocyte apoptosis.

### 2.3. A Positive Feedback Loop Exists between ER Stress Activation and ROS Generation Induced by FB1

**Figure 3:**
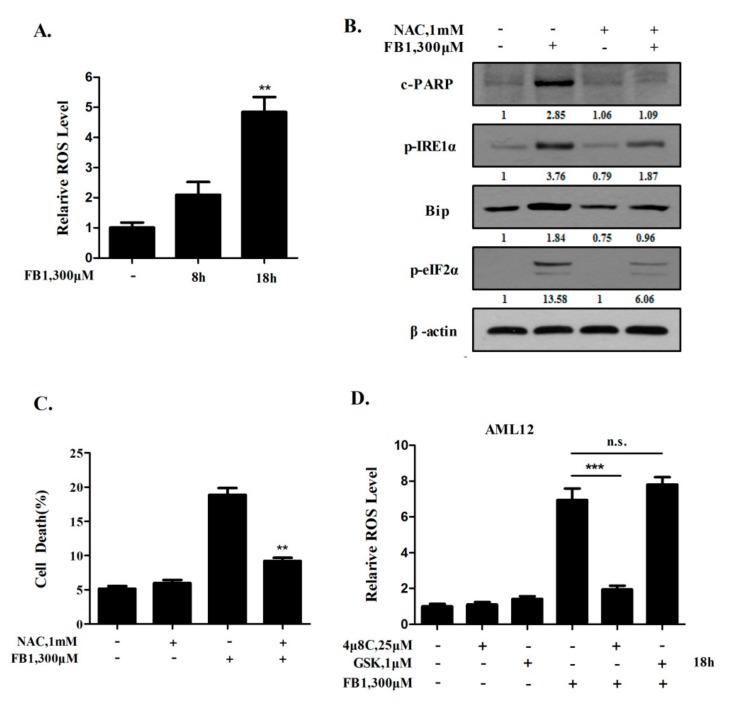
ROS generation is responsible for FB1-induced ER stress. (**A**) The effects of FB1 on the ROS generation. The cells were treated with FB1 for 8 h or 18 h, and the intercellular ROS levels were assessed using flow cytometry after DCFH-DA staining. (**B**) The influence of ROS suppression by NAC on the ER stress markers and cell apoptosis. The cells were treated with FB1 with or without NAC for 48 h, and the phosphorylation of IRE1α and eIF2α, Bip, and cleaved PARP were analyzed by Western blotting. n = 3. (**C**) The influence of ROS suppression by NAC on cell death induction. The cells were treated with FB1 with or without NAC for 48 h, and the cell death was evaluated by flow cytometry analysis after Annexin V staining. (**D**) The effect of the IRE1α or PERK specific inhibitor on FB1-induced ROS. The bars denote standard errors from three experiments. ** *p* < 0.01, *** *p* < 0.001 compared with the corresponding control.

It has been well documented that reactive oxygen species (ROS) generation and ER stress are closely linked events in apoptosis induction, and that these two cellular events can augment each other in a positive feedback loop under certain conditions [18]. Previous studies have shown that both oxidative stress and ER stress are induced by FB1 exposure [14,15,19,20]. We then investigated the relationship between FB1-induced ER stress and oxidative stress. AML12 cells were exposed to FB1 for the indicated time, and ROS was measured by flow cytometry following DCFH-DA staining. As shown in Figure 3A, treatment with FB1 induced a time-dependent increase of ROS in AML12 cells. To assess the role of the ROS generation in FB1-induced ER stress, we tested the effect of ROS suppression by N-acetyl-1-cysteine (NAC), a free radical scavenger and a precursor of glutathione, on FB1-induced key markers of ER stress. As shown in Figure 3B, the FB1-induced phosphorylation of IRE1α and eIF2α, and the induction of Bip, were significantly attenuated in the presence of NAC. In agreement with the ER stress inhibition by NAC, FB1-induced cell death (Figure 3C) and the cleavage of PARP (Figure 3B) were dramatically reduced under the condition of the ROS suppression. These results clearly indicate that ROS generation is an important contributor to the ER stress induction by FB1 exposure in liver cells. We next asked whether ROS-mediated ER stress led to a further ROS generation through a positive feedback mechanism. The changes of ROS levels were measured by flow cytometry when UPR signaling was inhibited by either the IRE1α or PERK inhibitor. As shown in Figure 3D, a significant reduction of FB1-induced ROS production was observed in the presence of the IRE1α inhibitor, whereas no significant change was detected in the presence of the PERK inhibitor. Together, these results suggest that a positive feed-forward loop existed between the ROS generation and IRE1α activation induced by FB1.

The disrupted sphingolipid metabolism-mediated accumulation of free sphingoid bases is another contributing factor for FB1-induced cytotoxicity. We next asked whether the ER stress induction was also associated with the disruption of the sphingolipid metabolism. Myriocin, a small molecule that blocks the sphingolipid biosynthesis pathway [21], was used to inhibit free sphinganine accumulation, and under such conditions neither the apoptosis induction nor ER stress activation was attenuated, ruling out the involvement of the accumulation of free sphingoid bases in the FB1-induced ER stress in liver cells (data not shown).

### 2.4. Inhibition of ER Stress by TUDCA Leads to a Significant Reduction of FB1-Induced Liver Injury In Vivo

**Figure 4:**
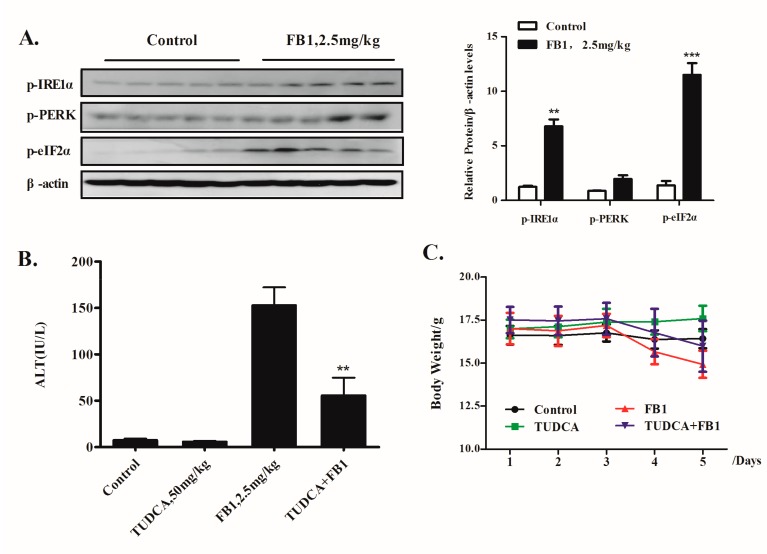
The inhibition of ER stress by TUDCA led to a significant reduction of FB1-induced liver injury in vivo. Animals and treatments are as described in Materials and Methods. (**A**) Western blot analysis of the expression of ER stress markers in the liver tissues of mice. n = 5. (**B**) The serum levels of ALT. (**C**) The body weight kinetics of mice. The quantitative data are presented as the mean ± SD based on biological repeats. ** *p* < 0.01, *** *p* < 0.001 compared with the corresponding control group.

Previous studies have established a correlation between hepatotoxicity and ER stress induction. However, it has not yet been addressed whether targeting ER stress can offer protection for FB1-induced liver injury in vivo. To investigate the role of ER stress in FB1-induced hepatotoxicity in vivo, we first confirmed ER stress induction in liver via FB1 exposure in a mouse model. As shown in Figure 4A, treatment with 2.5 mg/kgBW FB1 for 5 days caused a significantly increased phosphorylation of IRE1α, PERK, and eIF2α in liver samples. The elevated phosphorylation levels of these key ER stress-related proteins indicated that ER stress in liver was activated by FB1. To critically determine the functional role of ER stress in FB1-induced liver injury, we measured the effect of ER stress inhibition by its inhibitor tauroursodeoxycholic acid (TUDCA) on the level of alanine aminotransferase (ALT), a key biochemical marker of liver toxicity. As shown in Figure 4B, FB1 treatment induced a significant increase of serum ALT, which was dramatically reduced by TUDCA. Accordingly, the key ER stress markers were nearly completely abolished. In addition, FB1-induced body weight reduction was also attenuated by ER stress inhibition (Figure 4C). Together, the data suggest that FB1 exposure led to ER stress induction in liver, and that the inhibition of ER stress by its inhibitor significantly protected against FB1-induced liver injury.

### 2.5. Inhibition of ER Stress by TUDCA Inhibits FB1-Induced Hepatic Apoptosis and Autophagy In Vivo

**Figure 5:**
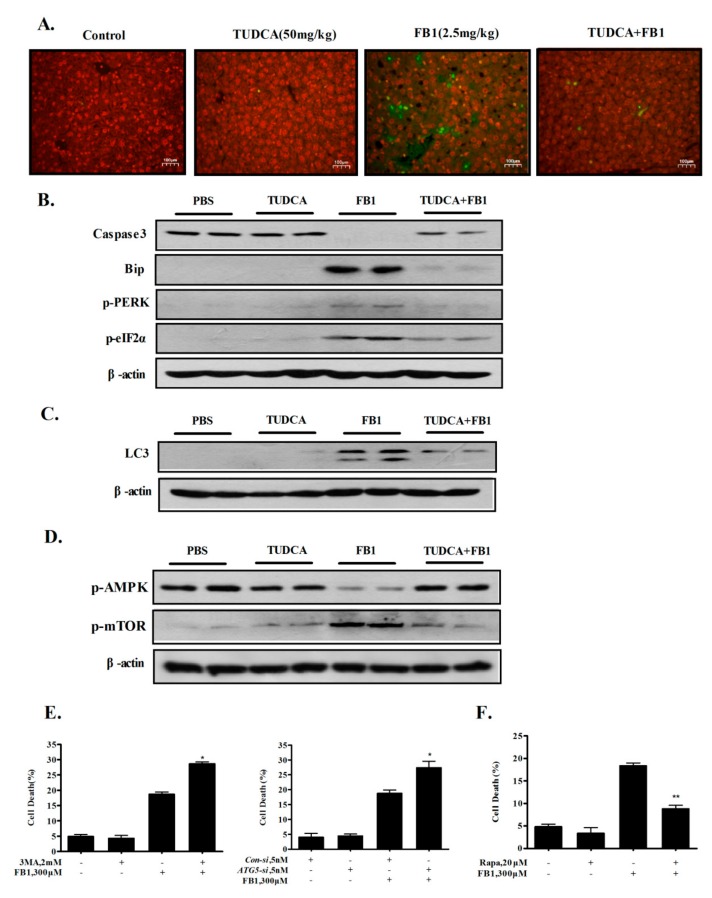
The inhibition of ER stress by TUDCA inhibited FB1-induced hepatic apoptosis and autophagy in vivo. (**A**) Hepatic apoptosis assessed by TUNEL. (**B**) A Western blot analysis revealed that FB1 induced the cleavage of caspase-3, which was rescued by ER stress inhibition in liver samples. (**C**) Western blot analysis of the LC3I/II expression. (**D**) Western blot analysis of p-AMPK, p-mTOR. (**E**) The influence of autophagy inactivation by the chemical inhibitor or RNAi on the apoptosis induction by FB1 in liver cells. (**F**) The effect of autophagy activation by rapamycin on the FB1-induced apoptosis in liver cells. The bars denote standard errors from three experiments. * *p* < 0.05, ** *p* < 0.01 compared with the corresponding control.

It has been shown that the induction of apoptosis and autophagy contributed to FB1-induced toxicity [14,22,23,24,25,26]. We next asked whether the inhibition of ER stress could protect against FB1-induced hepatic apoptosis and autophagy in vivo. Apoptosis was evaluated by a TUNEL assay detecting apoptotic DNA fragmentation and via a Western blot analysis of caspase-3. As shown in Figure 5A, FB1 exposure induced a significant increase in TUNEL-positive cells in liver samples, indicating that hepatic apoptosis was induced by FB1 under experimental conditions. As expected, FB1-induced hepatic apoptosis was significantly ameliorated by the ER stress inhibitor TUDCA. In agreement with the TUNEL assay results, a Western blot analysis revealed that the FB1 induced the cleavage of caspase-3, which was rescued by ER stress inhibition (Figure 5B). Autophagy was detected by a Western blot analysis of the LC3I/II conversion, which is a key marker of autophagy induction. As shown in Figure 5C, treatment with FB1 caused an increased LC3I/II conversion, which was suppressed by ER stress inhibition, suggesting a pivotal role of ER stress in activating hepatic autophagy by FB1 in vivo. We also investigated the involvement of the AMPK-mTOR axis in FB1-induced hepatic autophagy, and the results are shown in Figure 5D. The data showed that FB1 reduced AMPK phosphorylation and increased mTOR phosphorylation, indicating that FB1-induced autophagy was independent of AMPK activation or m-TOR inactivation. Taken together, the induction of ER stress, but not the inactivation of the mTOR pathway, played an important role in FB1-induced hepatic apoptosis and autophagy in vivo. To determine the relationship between apoptosis and autophagy in response to FB1, we examined the influence of autophagy inhibition by its inhibitor 3-MA or the knockdown of ATG5, or of autophagy induction by its inducer rapamycin on FB1-induced apoptosis. As shown in Figure 5E,F, apoptosis increased in the presence of 3-MA or by silencing ATG5, whereas a reduced apoptosis was observed in the presence of rapamycin, indicating that the activation of autophagy by FB1 counteracted its apoptotic effect in liver cells. Taken together, the data suggest that ER stress contributed to FB1-induced hepatic apoptosis and autophagy in vivo, and that the activation of autophagy by FB1 exposure acted as prosurvival signaling against apoptosis induction in liver cells.

### 2.6. Glycyrol Prevents FB1-Induced Apoptosis in AML12 Cells Through Inactivating IRE1α

**Figure 6:**
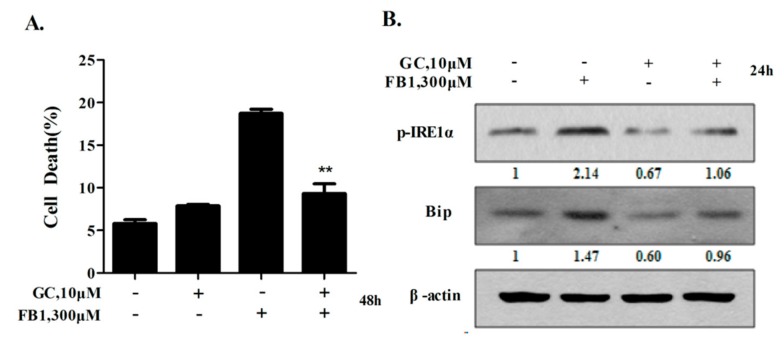
Glycyrol (GC) prevents FB1-induced apoptosis in AML12 cells through inactivating IRE1α. The effects of GC on FB1-induced cell death. (**A**) The cells were exposed to 300 μM FB1 with or without 10 μM GC for 48 h, and then the cells were collected for a death measurement by Annexin V/PI staining. The bars denote standard errors from three experiments. (**B**) The influences of GC on the FB1-mediated ER stress. The cells were exposed to 300 μM FB1 with or without 10 μM GC for 24 h, and then IRE1α phosphorylation and Bip were examined by Western blotting. n = 3. ** *p* < 0.01 compared with the corresponding control.

Licorice, a popular edible and medicinal plant, has been demonstrated to possess multiple biological activities, including a hepatoprotective effect [27,28]. Glycyrol (GC) is a naturally occurring plant coumarin compound isolated from licorice [29]. Our previous studies have shown that glycycoumarin (GCM), an analogue of GC, protected against hepatotoxicity in multiple model systems through mechanisms involved in the suppression of ER stress [30]. We then examined if GC could offer protection for FB1-induced apoptosis through inactivating IRE1α. AML12 cells were exposed to FB1 with or without GC for 48 h, and apoptosis was analyzed by Annexin V/PI staining. As demonstrated in Figure 6A, apoptosis induced by FB1 was significantly reduced by GC. In line with the decreased apoptosis, FB1-induced IRE1α phosphorylation and Bip up-regulation were obviously suppressed by GC (Figure 6B). The data suggest that GC was capable of protecting liver cells from FB1-induced apoptosis via the inactivation of IRE1α.

## 3. Discussion

Liver is major target organ site of FB1 toxicity, and the activation of ER stress is considered to be a critical event for FB1-induced hepacytotoxicity. In the present study, we further deciphered the mechanisms underlying ER stress-triggered liver toxicity in response to FB1 exposure. We demonstrated that the activation of the IRE1α-mitochondria pathway but not of the PERK axis of the ER stress response contributed to FB1-induced ER stress-mediated hepatocyte toxicity. Moreover, our data revealed that FB1-induced ROS generation and ER stress induction accentuated each other through a positive feed-forward loop. In addition, we provided evidence that inactivating ER stress via its inhibitor led to a significant reduction of FB1-induced hepatotoxicity in vivo. The data enable us to better understand the functional role of ER stress in FB1-induced hepatotoxicity.

When ER stress is induced, a number of intracellular signal transduction pathways are activated. To date, at least three mechanistically distinct arms of the UPR signaling (PERK, IRE1a, and ATF6) have been identified. Among the UPR signaling pathways, IRE1α and PERK are two key molecules that regulate the cell fate of ER-stressed cells. The activation of IRE1α and PERK can exert an either protective or pro-death function depending on the context [2,15]. Both IRE1α and PERK were activated by FB1 exposure. Regarding the functional role of these two branches of UPR, in the current study we uncovered that the inhibition of IRE1α, but not of PERK, protected the liver from FB1-induced toxicity. Accordingly, inactivating IRE1α by its specific inhibitor led to nearly abolishing the activation of JNK and the mitochondrial pathway induced by FB1. The data therefore supported that the FB1-induced ER stress-mediated hepatotoxicity was attributed to the activation of the IRE1α-JNK-mitochondria pathway but not to PERK. The data of the current study provided novel insight into the mechanistic understanding of the hepatotoxicity induced by FB1.

Autophagy can be induced by various types of stimuli, including mycotoxins [14]. The activation of autophagy can either protect against cell death induction or promote cell death induction, depending on the context [31]. The determinants that govern the pro-death or pro-survival function of autophagy remain elusive. The proposed factors that affect the functional role of autophagy in regulating cell death induction include the magnitude and duration of autophagy or the types of cells [32]. Our previous study has shown that treatment with FB1 dose-dependently activated autophagy in MARC-145 monkey kidney cells. The inhibition of autophagy by either RNAi or chemical inhibitors resulted in a significantly reduced cell death, suggesting that the autophagy induction by FB1 contributed to the cell death induction in MARC-145 monkey kidney cells [14]. Consistent with the pro-death function of autophagy in MARC-145 monkey kidney cells, a recent study by Zhang et al. [33] demonstrated that FB1 can cause autophagic cell death in the hemocytes of *Ostrinia furnacalis*. In the present study, we showed that hepatic autophagy was activated by FB1 both in vitro and in vivo. The inhibition of autophagy by its inhibitor or the RNAi approach led to an increased cell death induction in AML12 mouse liver cells, while the autophagy inducer rapamycin protected the liver cells from FB1-induced cell death. These results clearly indicated that hepatic autophagy induction exerted a pro-survival activity against FB1-induced hepatocytotoxicity, which is consistent with that found in HepG2 cells [15]. An obvious explanation for this controversial role of FB1-induced autophagy in regulating cell death induction is the types of cells. The detailed mechanisms involved in the pro-death or pro-survival function of FB1-induced autophagy need to be further investigated. Moreover, the validation of these in vitro findings in vivo is also needed.

## 4. Conclusions

The activation of IRE1α, but not of the PERK branch, of the ER stress response contributed to FB1-induced ER stress-mediated hepatocyte toxicity. The hepatic ER stress activation by FB1 was attributed to the oxidative stress, not to the accumulation of free sphingoid bases, and FB1-induced ER stress promoted ROS generation through a positive feedback mechanism. The suppression of ER stress by its chemical inhibitor could offer protection against FB1-induced liver toxicity in vivo. The findings of the present study provided novel insight into understanding the mechanisms underlying FB1-induced ER stress-mediated liver toxicity, and strongly suggested that targeting ER stress is a practical and an effective approach for fighting against FB1-mediated liver toxicity in vivo.

## 5. Materials and Methods

### 5.1. Chemicals and Reagents

Fumonisin B1 and Tauroursodeoxycholic acid (TUDCA) were purchased from Cayman Chemical (Ann Arbor, MI, USA). Glycyrol (GC purity > 99%) was purchased from BioBioPha (Kunming, Yunnan, China). 3-methyladenine (3-MA), bafilomycin A1, *N*-acetyl-L-cysteine (NAC), Tris-HCl, and DCFH-DA were purchased from Sigma-Aldrich (St. Louis, MO, USA). ISP-1 (476300) was purchased from Calbiochem (San Diego, CA, USA). IRE1α inhibitor 4μ8C and PERK inhibitor GSK2606414 were purchased from MCE (Shanghai, China). Primary-antibodies specific to caspase-3 (9662), Bip (3183), phospho-eIF2α (3597), phospho-PERK (3192), CHOP (2895), Bax(2772), Bak (12105), BCl-2 (3869), PUMA (14570), c-PARP (9548), p-AMPK (2535), p-mTOR (2448), Mcl-1 (4572), and p-JNK (4668) were purchased from Cell Signaling Technology. Phospho-IRE1α (ab48187) was purchased from Abcam (Beverly, MA, USA). Antibody for LC-3 was purchased from MBL International Corporation (Woburn, MA, USA). β-actin antibody was purchased from Action Biotech. Rabbit (458) and Mouse (330) second antibodies were purchased from MBL International Corporation. The primary antibody dilution ratio is 1:1000. The dilution ratio of second-antibody specific for either rabbit or mouse is 1:5000. Protease inhibitor cocktail (AEBSF, hydrochloride; aprotinin; E-64 protease inhibitor; EDTA, disodium salt; leupeptin hemisulfate. Cat. No. 539131) was purchased from Calbiochem.

### 5.2. Cell Culture and Treatments

AML12 mouse liver cells were obtained from the American Type Culture Collection (ATCC) and grown in DMEM/F12 medium supplemented with 10% fetal bovine serum and 1% ITS without antibiotics. MEF mouse embryonic fibroblast cells (generously provided by Professor Feng Zhu, Tongji Medical College, Huazhong University of Science and Technology) were grown in DMEM medium supplemented with 10% fetal bovine serum without antibiotics. Treatments were given when the cell confluency reached around 50–60%.

### 5.3. Apoptosis Evaluation

Apoptosis was evaluated by the Annexin V staining of externalized phosphatidylserine in apoptotic cells by flow cytometry using a commercially available kit (MBL International, Woburn, MA, USA). Briefly, the cells were treated for the times indicated. After the treatments, the cells were harvested and washed twice with ice-cold PBS, and re-suspended in a 1× binding buffer. Subsequently, the cells were incubated with Annexin V-FITC staining solution at room temperature for 15 min, and analyzed by Becton Dickinson FACSCalibur Flow Cytometer at an excitation wavelength of 488 nm. Ten thousand cells were collected from the analyzed sample, the cells of Annexin V positive and PI negative represented early apoptotic cells, the cells of both Annexin V and PI positive represented late apoptotic cells, and the cells positive for PI only represented necrotic cells. The percentage of cell deaths was calculated by adding up early apoptotic cells, late apoptotic cells, and necrotic cells, and dividing the total cell number.

### 5.4. Proliferation Assay

The proliferation was evaluated by crystal violet staining. After the treatments, the culture medium was removed, and 1% glutaraldehyde solution was used to fix the cells for 15 min. After the fixation, 0.02% aqueous solution of crystal violet was used to stain the cells for 30 min. After washing with PBS, the stained cells were solubilized with 70% ethanol. The absorbance at 570 nm with the reference filter 405 nm was assessed by a microplate reader (Thermo, MK3, Waltham, MA, USA).

### 5.5. Western Blotting

Western blot analyses were essentially conducted as described previously [34]. Briefly, ice-cold RIPA (radio-immuno-precipitation assay) buffer containing protease inhibitor was used to lyse the cells. Proteins of the samples were separated by electrophoresis and then transferred to a nitrocellulose membrane (PALL, Pensacola, FL, USA). The membrane was subsequently incubated with primary antibodies following the incubation with secondary antibody. The immunoreacted bands were visualized by enhanced chemiluminescence (Fisher/Pierce, Rockford, IL, USA) and recorded on an X-ray film (Eastman Kodak Company, Rochester, NY, USA; XBT-1).

### 5.6. Assessment of Reactive Oxygen Species

AML12 cells were treated with FB1 for the times indicated. 30 min before the cell harvest, 20 μM DCFH-DA (2′,7′-dichlorodihydrofluorescein diacetate) was added to the cultured medium. Esterases can hydrolyze DCFH-DA to DCFH, which is then oxidized by hydrogen peroxides to generate fluorescent DCF. The enhanced intracellular fluorescence was assessed by a 530 nm bandpass filter with a Becton Dickinson FACSCalibur Flow Cytometer. The ROS scavenger NAC was added 2 h before the treatment of FB1.

### 5.7. RNA Interference

siRNAs targeting ATG7 (41447), and non-targeting siRNA (37007) were obtained from Life Technologies. The cells were transfected with siRNAs using siPORT NeoFX transfection agent (AM4510). 24 h post-transfection, the cells were used for subsequent experiments.

### 5.8. Animals and Treatments

Eight-week-old male C57BL/6N mice weighing 20.0 ± 1.0 g were purchased from Vital River (Beijing, China). Animal care and procedures were approved by the Institutional Animal Care and Use Committee (China Agricultural University). Approval code: 3197190316; approval date: 11 March 2019. The mice were fed with a commercial standard mouse cube diet (Beijing Keaoxieli Feed Company, Beijing, China). After acclimatization for 5 days, the mice were randomly divided into 4 groups, and each group contained 7 mice. Group 1: vehicle control with the injection of physiological saline. Group 2: TUDCA (50 mg/kg, i.p. treatment for 7 days) according to the previous studies [35,36]. Group 3: FB1 (2.5 mg/kg, i.p. treatment for 5 days) according to the previous studies [15]. Group 4: TUDCA (50 mg/kg, i.p. treatment for 7 days) and FB1 (2.5 mg/kg, i.p. treatment for 5 days). The mice were treated with TUDCA two days prior to the FB1 treatment, and then treated with FB1 and/or TUDCA every day for 5 days continuously. FB1 and/or TUDCA were dissolved in physiological saline. The mice were sacrificed 24 h after the last injection. The liver tissues were collected immediately for the following research. The liver tissues were either fixed in neutral buffered formalin or frozen in liquid nitrogen immediately.

### 5.9. ALT Measurement

The serum alanine aminotransferase (ALT) activity was determined by measuring the enzyme reaction-mediated production of colorimetric product using a commercially available ALT activity assay kit from Nanjing Jiancheng (Nanjing, China), according to the manufacturer’s instructions.

### 5.10. Histochemical and Immunohistochemical Staining

Apoptosis in mouse liver tissues was evaluated via a Terminal deoxynucleotidyl transferase-mediated dUTP nick end labeling assay (DeadEnd™ Fluorometric TUNEL System, Promega Corporation, WI, USA), according to the manufacturer’s instructions.

### 5.11. Statistical Analysis

The data are presented as the mean ± SD. The statistical analysis was carried out via a one-way ANOVA followed by Tukey’s post hoc test using SPSS20.0. The graphs were drawn using GraphPad Prism (version 5.0 for MacOS, La Jolla, CA, USA, 2014)
